# Systemic Toll-Like Receptor Stimulation Suppresses Experimental Allergic Asthma and Autoimmune Diabetes in NOD Mice

**DOI:** 10.1371/journal.pone.0011484

**Published:** 2010-07-07

**Authors:** Aude Aumeunier, Françoise Grela, Abdulraouf Ramadan, Linh Pham Van, Emilie Bardel, Alejandro Gomez Alcala, Pascale Jeannin, Shizuo Akira, Jean-François Bach, Nathalie Thieblemont

**Affiliations:** 1 Université Paris Descartes, Paris, France; 2 CNRS, UMR8147, Paris, France; 3 INSERM U564, Angers, France; 4 Department of Host Defense, Osaka University, Osaka, Japan; 5 INSERM U1013, Paris, France; Fundação Oswaldo Cruz, Brazil

## Abstract

**Background:**

Infections may be associated with exacerbation of allergic and autoimmune diseases. Paradoxically, epidemiological and experimental data have shown that some microorganisms can also prevent these pathologies. This observation is at the origin of the hygiene hypothesis according to which the decline of infections in western countries is at the origin of the increased incidence of both Th1-mediated autoimmune diseases and Th2-mediated allergic diseases over the last decades. We have tested whether Toll-like receptor (TLR) stimulation can recapitulate the protective effect of infectious agents on allergy and autoimmunity.

**Methods and Findings:**

Here, we performed a systematic study of the disease-modifying effects of a set of natural or synthetic TLR agonists using two experimental models, ovalbumin (OVA)-induced asthma and spontaneous autoimmune diabetes, presenting the same genetic background of the non obese diabetic mouse (NOD) that is highly susceptible to both pathologies. In the same models, we also investigated the effect of probiotics. Additionally, we examined the effect of the genetic invalidation of MyD88 on the development of allergic asthma and spontaneous diabetes. We demonstrate that multiple TLR agonists prevent from both allergy and autoimmunity when administered parenterally. Probiotics which stimulate TLRs also protect from these two diseases. The physiological relevance of these findings is further suggested by the major acceleration of OVA-induced asthma in MyD88 invalidated mice. Our results strongly indicate that the TLR-mediated effects involve immunoregulatory cytokines such as interleukin (IL)-10 and transforming growth factor (TGF)-β and different subsets of regulatory T cells, notably CD4^+^CD25^+^FoxP3^+^ T cells for TLR4 agonists and NKT cells for TLR3 agonists.

**Conclusions/Significance:**

These observations demonstrate that systemic administration of TLR ligands can suppress both allergic and autoimmune responses. They provide a plausible explanation for the hygiene hypothesis. They also open new therapeutic perspectives for the prevention of these pathologies.

## Introduction

There is compelling evidence to indicate a central role of Toll-like receptors (TLRs) in the stimulation of innate and adaptive immunity when applied at the site of immune responses [1]–[3]. More limited but convincing observations suggest a possible role of TLRs in the triggering of allergic and autoimmune diseases [4]–[9]: such induction could not be obtained in several experimental models after genetic invalidation of certain TLRs or one of their adaptor molecules, namely MyD88 [6], [7], [10], [11]. On the other hand, more unexpected observations have indicated that systemic TLR stimulation can prevent the onset of allergic and autoimmune diseases when it is implemented early enough in the natural history of the disease. Thus, administration of the TLR4 agonist LPS and of the TLR9 agonist CpG has been shown to prevent spontaneous diabetes onset in the non obese diabetic (NOD) mouse [12], [13]. Similarly, administration of various TLR agonists may prevent onset of ovalbumin (OVA)-induced allergic asthma in various mouse strains including BALB/c and A/J mice [14]–[20], even though the same agonists may exceptionally show the opposite effect depending on the experimental conditions [21]–[24].

TLR-mediated prevention of allergic and autoimmune diseases could represent one of the major mechanisms underlying the hygiene hypothesis according to which the major increase of these diseases observed in western countries over the last three decades is secondary to the decline of infections [25]–[27]. Originally elaborated in the context of an increased susceptibility to allergy we proposed back in 2002 that the hypothesis could also apply to autoimmune diseases [28]. Validating the hygiene hypothesis in the clinical setting is a complex issue. A major problem is that some particular infections may trigger/exacerbate either allergic or autoimmune diseases and that the nature of the infections contributing to protection is still ill-defined. This is why so far the best direct evidence in support of the hygiene hypothesis has been collected from experimental animal models such as the NOD mouse in which a variety of pathogens (living pathogens or bacterial extracts) totally prevent autoimmune diabetes onset [29]–[32] (reviewed in [28]). The study of bacterial extracts, which are easier to use and analyze as compared to living pathogens, is complicated by the multiplicity of their components.

When reports on the hygiene hypothesis were confined to allergic diseases the Th1/Th2 paradigm was proposed as a leading mechanism to explain the effect observed. Thus, given the reciprocal down-regulation of Th1 and Th2 cells some authors initially suggested that, in developed countries, the lack of microbial burden in early childhood which normally favors a strong Th1-biased immunity would redirect the immune response towards a Th2 phenotype and, therefore, predispose the host to allergic disorders. Such conclusions were challenged once the hygiene hypothesis was extended to autoimmunity as Th1 responses in the case of autoimmunity are not protective but pathogenic [28].

Taking into account these considerations we chose to study the effect of TLR stimulation in the NOD mouse that is an experimental model where development of allergy and spontaneous autoimmunity can be studied in parallel in the context of an identical genetic background. In fact, NOD mice represent a particularly appropriate model since, in addition to their well documented susceptibility to develop spontaneous autoimmune diabetes [33], [34], they have been shown to be a strain that is highly susceptible to the induction of allergic asthma [35]. It was our rationale that this approach would provide us with the opportunity to test whether common regulatory immune mechanisms, possibly involving TLR stimulation, exist underlying infection-mediated protection against both allergy and autoimmunity namely, whether these common regulatory immune mechanisms control both Th1 and Th2 responses.

Thus, one may postulate that TLR ligands that are present in numerous pathogens have a general non specific inhibitory effect on allergic and autoimmune responses.

Here, we have tested whether TLR stimulation can recapitulate the protective effect of infectious agents on allergy and autoimmunity. To that aim we first performed a systematic screening of a set of natural or synthetic TLR agonists in two experimental models, OVA-induced asthma and autoimmune diabetes. Secondly, in the same models we investigated the effect of probiotics. We also examined the effect of the genetic invalidation of MyD88 on the development of spontaneous diabetes, as previously performed by the group of A. Chervonsky [36] and additionally in the allergic asthma model.

Collectively, results presented in this study demonstrate that systemic TLR stimulation by both probiotics and TLR agonists is efficacious in preventing from both allergy and autoimmunity.

## Materials and Methods

### Mice

Conventional NOD mice (K^d^, I-A^g7^, and D^b^) were bred in our animal facility at the Hôpital Necker as well as CD28^−/−^NOD mice (a kind gift from J.A. Bluestone, UCSF, San Francisco, CA), CD1d^−/−^NOD mice (a kind gift from M. Kronenberg, La Jolla institute, San Diego, CA) and IL-4^−/−^NOD mice (a kind gift from D. Mathis and C. Benoist, Josslin Center, Boston, MA). We backcrossed MyD88^−/−^C57BL/6 mice into the NOD genetic background (N13). Seven-week-old female C57BL/6 mice were purchased from Janvier.

### Ethics Statement

All experiments have been conducted in accordance with European Union Council Directives (86/609/EEC) and with institutional guidelines (INSERM: Institut National de la Santé et de la Recherche Médicale). The animal facility has an agreement delivered by the *Prefecture de Police* of Paris, France.

### TLR agonists and probiotics

The P40 protein of *Klebsiella pneumoniae* was purified as previously described [37]. We obtained purified lipopolysaccharide (LPS) from *S. minnesota* and R848 (resiquimod) from Alexis Biochemicals (Paris, France). Polyinosinic-polycytidylic acid (Poly(I∶C)) was purchased from Invivogen (Toulouse, France) and Sigma-Aldrich (St Louis, MO) as well as lipid A from *S. Minnesota*. Pam_3_Cys: *S*-[2,3-bis(palmitoyloxy)-(2-RS)-propyl]-*N*-palmitoyl-(R)-Cys-(S)-Ser-Lys4-OH from Invivogen. The probiotic preparation VSL#3 containing *bifidobacterium, lactobacillium* and *streptococcus* was purchased from Sigma-tau (Ivry-sur-Seine, France).

### The ovalbumin-induced airway inflammation model and treatments

On day 0 NOD mice were sensitized with 100 µg of chicken egg OVA (Sigma-Aldrich) in 1.6 mg aluminium hydroxide *i.p.* in a volume of 200 µl. Then they were challenged with 50 mg/ml OVA upon aerosol exposure on three consecutive days (days 7–9) to induce allergic airway inflammation. Controls received a NaCl solution. The TLR agonists P40, Poly(I∶C), LPS or R848 were injected *i.p*. 24 hrs and 1 hr before the first challenge.

We assessed lung function using two different methods. The first was non-invasive barometric plethysmography and consisted in measuring airway hyper-responsiveness (AHR) 24 hrs after the last OVA challenge by delivering an aerosol of methacholine (Mch) (Sigma-Aldrich) for 1 min at 150 mM to mice placed in a plethysmographic chamber (EMKA technologies, Paris, France). The index of airflow obstruction was expressed as enhanced pause (Penh). In some experiments airway resistance and compliance were measured with the FlexiVent device (FlexiVent; SCIREQ, Montreal, Québec, Canada). Twenty four hours after the last challenge, airway resistance (R) and compliance (C) to methacholine were measured. Mice were anesthetized with an intraperitoneal injection of sodium pentobarbital (70 mg/kg). The trachea was exposed, a tracheotomy was performed to insert a 18-gauge needle connected to a computer-controlled small-animal ventilator. Mice were quasi-sinusoidally ventilated with a tidal volume of 10 ml/kg at a frequency of 150 breaths/min and a positive end-respiratory pressure of 2 cmH_2_O to achieve a mean lung volume close to that during spontaneous breathing. After recording baseline values, each mouse was challenged with an aerosol of methacholine, generated with an in-line nebulizer and administered directly through the ventilator for 5 seconds delivering increasing concentrations (0, 0.625, 1.25, 2.5, 5, and 10 mg/ml). R and C were measured with a “snapshot” protocol each 20 seconds for 2 min. The mean of these six values was deduced for each methacholine concentration. For each mouse, R and C were plotted against methacholine concentration (from 0 to 10 mg/ml), as previously described [38].

It is relevant to mention that in our colony NOD mice develop spontaneous insulin-dependent diabetes as assessed by hyperglycemia and glycosuria by 12–13 weeks of age. Before this point in time all animals exhibit normal glucose levels. As done in other strains OVA sensitization was performed in 8 week-old mice and given the kinetics of the experiment (described above) the challenge was performed one week later (day 7 to 9). Therefore experiments were completed when NOD mice were 9–10 week old which explains that we never observed abnormal glucose levels in NOD mice undergoing OVA sensitization/challenge.

For probiotic treatment, VSL#3 preparation (5. 10^9^ bacteria/mouse in 100 µl PBS) was administered by gavage 5 days a week during 6 weeks before the OVA immunization. In these experiments control mice were treated with PBS.

An infraoptimal protocol was also set for MyD88^+/+^ and MyD88^−/−^ NOD mice. Here mice were immunized with only 50 µg of OVA in 1.6 mg of aluminium hydroxide and challenged by aerosol exposure with a single reduced dose of OVA (20 mg/ml) on day 7. Samples were collected 48 hrs after the challenge for further analyses.

In some experiments a monoclonal antibody specific for the IL-10 receptor, that neutralizes IL-10 activity (1B1.2) was administered *i.p.* 48 hrs and 1 hr before the first challenge.

For adoptive transfer experiments, 5×10^6^ CD4^+^ cells were purified from total spleen cells following magnetic bead sorting (Myltenyi Biotec, Paris, France) recovered from either probiotic-treated or untreated control NOD mice. The cells were injected *i.v.* to syngeneic recipients that had already been immunized with OVA and which received the CD4^+^ cell infusion 1 hr before the first challenge.

### Bronchoalveolar lavage

Mice were euthanized with urethane (Sigma-Aldrich) administered *i.p*. The lungs were cannulated through the trachea to perform the BALF. Cellular fractions were recovered and processed for differential staining by cytospin centrigugation. The BALF as well as lung homogenates were analyzed for cytokine and chemokine content by ELISA (R&D Systems, Lille France) according to manufacturers' specifications.

### Monitoring for autoimmune diabetes and treatment of NOD mice

We treated NOD mice with TLR agonists or PBS and monitored weekly for clinical signs of diabetes using Gluko-Test reagent sticks, to detect glucose in urine samples (Boehringer Mannheim, Meylan, France). When needed, glycemia was also measured in a drop of blood collected from the tail vein and using a Reflolux S glucometer (Boehringer Mannheim). Incidence of diabetes was defined based on the discovery, upon serial monitoring, of glycosuria and hyperglycemia (fasting glycemia >2.5 g/L).

NOD mice were also treated with probiotics (VSL#3; 5.10^9^ bacteria/mouse in 100 µl PBS) delivered orally by gavage 5 times a week starting at 4 weeks of age. In these experiments control mice received PBS.

### Histological analysis

Pancreas were collected when needed, fixed in 4% formaldehyde and paraffin-embedded. Serial 5-µm sections were stained with hematoxylin and eosin. Mononuclear cell infiltration was scored by counting at least 100 independent islets/recovered pancreas and distinguishing three distinct patterns that were: 1) Intact islets: islets showing a normal morphology and total absence of infiltrating mononuclear cells, 2) Peri-insulitis: islets showing a generally preserved morphology but presenting a significant number of mononuclear cells that remain confined to the periphery of the islets and 3) Destructive insulitis: islets showing a disrupted morphology and a significant number of invading mononuclear cells.

### *In vitro* cultures

For cytokine production 2×10^5^ splenocytes from MyD88^+/+^ and MyD88^−/−^ C57BL/6 mice were cultured in complete medium: RPMI supplemented with antibiotics and 10% fetal calf serum (Invitrogen, Cergy-Pontoise, France) in the absence or presence of TLR agonists at varying doses. After 48 (for IL-10) to 72 (for TGF-β) hrs of culture at 37°C, supernatants were harvested. For TGF-β, responses to TLR agonists were compared to control cultures performed in serum-free medium. When needed peritoneal macrophages were collected 48 hrs following *i.p.* injection with 2 ml of thioglycollate broth (BioMérieux, Craponne, France). Macrophages were then cultured in presence of probiotics for 24 hrs at 37°C. All supernatants were harvested and stored at −80°C until cytokines were dosed by ELISA (R&D systems, Lille, France).

### Circulating cytokine analysis

NOD mice were injected with TLRs agonists or fed with probiotics for two weeks (see details in the results section). Twenty four hrs after the end of treatment, serum samples were collected and levels of circulating TGF-β and IL-10 were measured by ELISA (R&D systems).

### Flow cytometry analysis

Spleen cells were recovered following treatment with TLR agonists or probiotics (see details in the results section). Cell suspensions were stained with antibodies to CD25 (labeled to phycoerythrin (PE)) and CD4 (labelled to phycoerythrin fluorescein isothiocyanate (FITC)) (BD Biosciences, Pont de Claix, France). Then cells were fixed and labelled with the Foxp3 kit (BD Biosciences) according to manufacturer's instructions. Samples were collected on a FACSCantoII cytometer (BD Biosciences). Data were gated on mononuclear cells with forward- and side-scatter properties using the FACS Diva Software.

### Statistical analysis

Diabetes incidence was plotted using the Kaplan-Meier method, i.e., nonparametric cumulative survival plot. Statistical comparison between curves was performed using the logrank (Mantel-Cox) test that provided the corresponding χ^2^ values. When needed, statistical comparison of mean values was performed using Student's t test. In the allergic model, the difference between groups was calculated with the Mann-Whitney *U* test for unpaired data (GraphPad Prism Software, La Jolla, CA). Differences were considered significant when *P*<0.05 (* *P*<0.05, ** *P*<0.01, *** *P*<0.005).

## Results

### Effect of TLR agonists on allergic asthma and autoimmune diabetes in NOD mice

**Figure 1:**
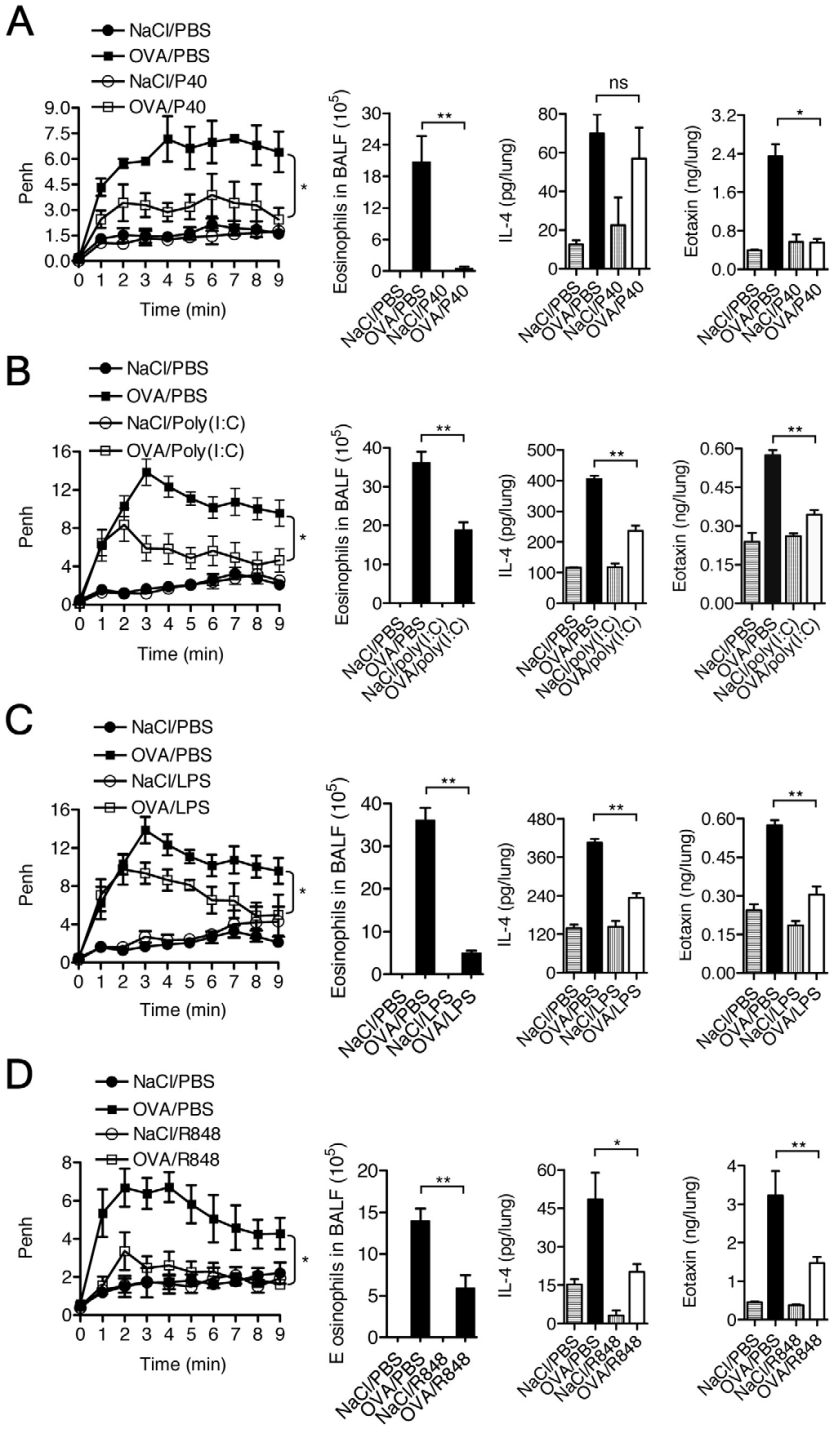
Stimulation of TLR pathways prevents allergic inflammation and airway hyper-responsiveness. NOD mice were treated as described in the Methods section. Briefly, mice immunized with OVA on day 0 were challenged with OVA or NaCl (controls) on days 7, 8 and 9. Each TLR agonist or phosphate-buffered saline (PBS) was administered 24 hrs and 1 hr before the first challenge. OVA-challenged mice were treated with PBS or (**A**) P40 (200 µg/challenge/mouse), a TLR2 agonist, (**B**) Poly(I∶C) (100 µg/challenge/mouse), a TLR3 agonist, (**C**) LPS (100 µg/challenge/mouse), a TLR4 agonist and (**D**) R848 (100 µg/challenge/mouse), a TLR7 agonist. AHR to Mch was measured 24 hrs after the last challenge and total cell as well as eosinophils in BALF and cytokine and chemokine concentrations in lungs. Mice treated with TLR agonists showed a decreased AHR, eosinophilia and IL-4 and eotaxin production as compared to control mice (* p<0.05; ** p<0.01). These experiments were performed twice using 4 to 6 mice per group. One representative experiment is shown.

**Figure 2:**
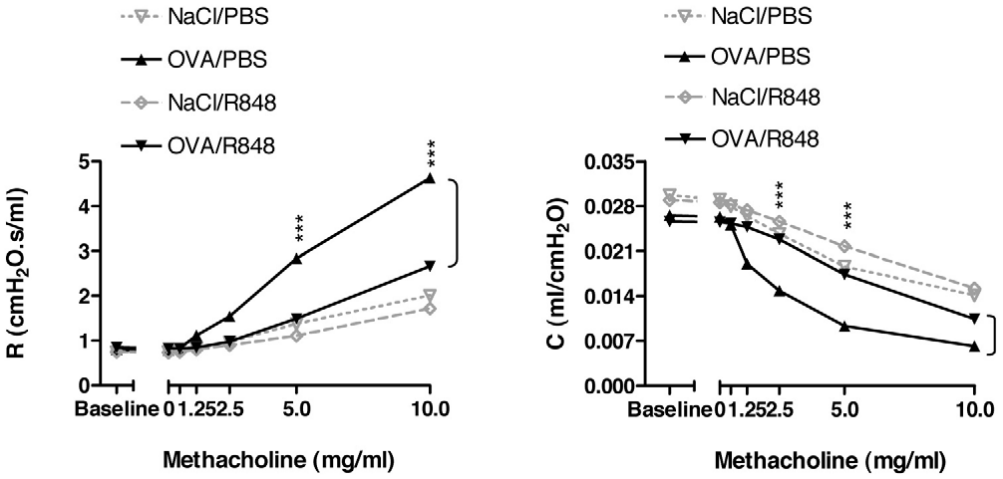
Treatment with the TLR7 agonist R848 prevents airway resistance and compliance. NOD mice immunized with OVA were injected with R848 and challenged with OVA as described in the methods section. Lung resistance (R) and compliance (C) were measured 24 hrs after the last aerosol challenge with OVA or NaCl. Results are representative of one experiment out of 2 performed using 4 to 6 mice per group.

We tested the effect of agonists of TLR2 (P40 protein of *Klebsiella pneumoniae* and the Pam_3_Cys lipopeptide), TLR3 (double-stranded RNAs Poly(I∶C)), TLR4 (LPS and lipid A) and TLR7 (R848) on experimental allergic asthma and autoimmune diabetes in the NOD mouse. In the asthma model, NOD mice were immunized intraperitoneally on day 0 with OVA in presence of alum, challenged one week later with 3 consecutive OVA aerosol administrations and analyzed 24 hrs after the last challenge. Following this protocol, mice presented allergic inflammation and abnormal lung function. Allergic inflammation resulted in an increase of cell recruitment including eosinophils in the BALF, and of cytokine and chemokine production, IL-4 and eotaxin respectively, in the lung. Lung function was assessed by measuring AHR using non-invasive whole-body plethysmography (Figure 1) and also in some experiments by measuring airway resistance (R) and compliance (C) using an invasive method (Figure 2).

The TLR2, TLR3, TLR4 and TLR7 agonists were administered 24 hrs and 1 hr before the first challenge. In treated mice a significant decrease in AHR as well as in eosinophil counts in BALF and levels of IL-4 and eotaxin in the lung were observed (Figure 1). Levels of IL-5, IL-13, IL-17 and TARC in the lungs were also decreased (data not shown). As measurement of AHR using non-invasive plethysmography may be subject biases (linked to changes in mice breathing patterns) we validated our results, in the case of mice treated with the TLR7 agonist (R848) by measuring airway resistance and compliance using a FlexiVent device. As detailed in Figure 2, R848 administration resulted in significantly decreased lung resistance and compliance which confirmed the AHR values previously described (Figure 1).

**Figure 3:**
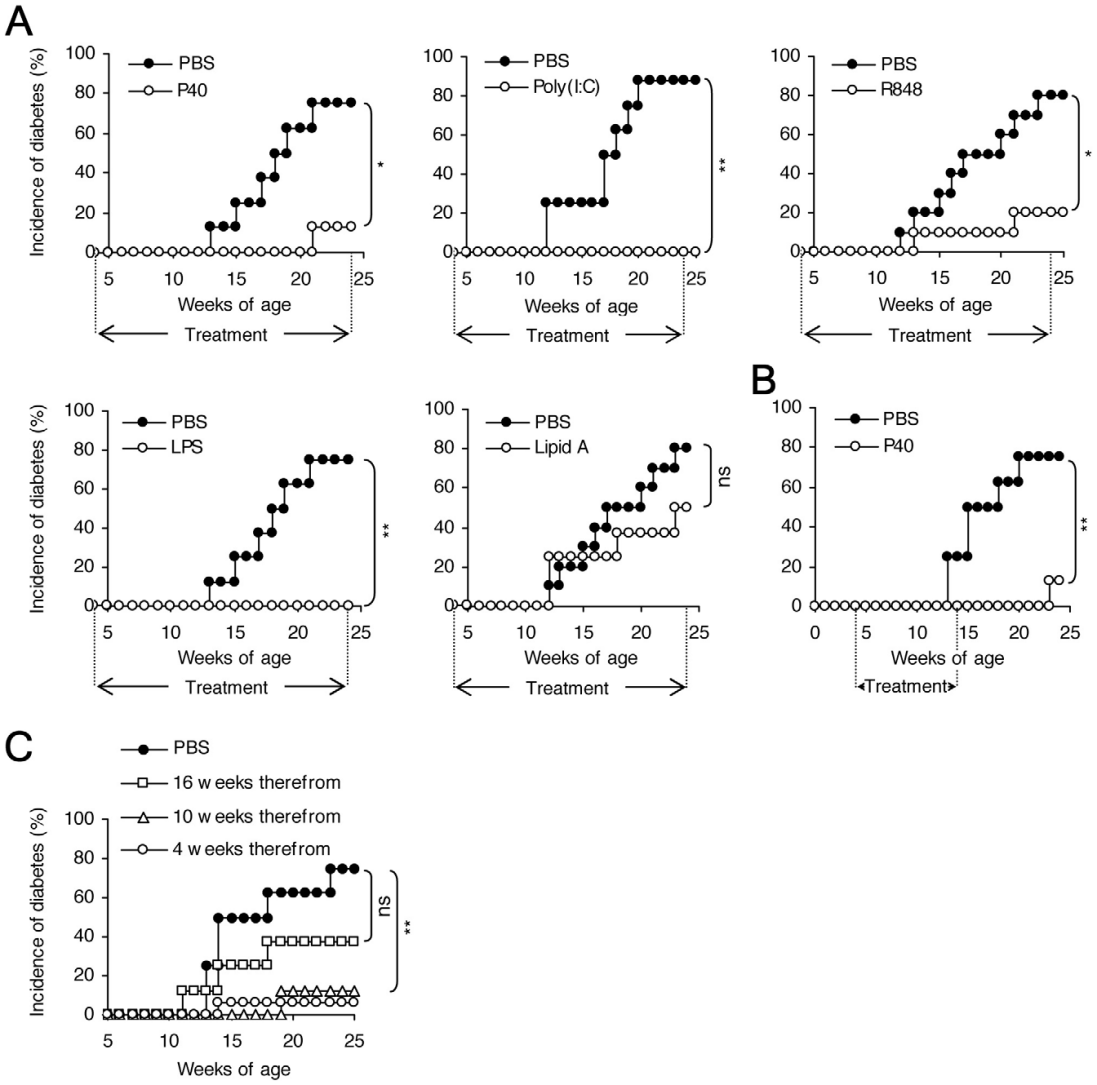
Stimulation of TLR pathways prevents spontaneous autoimmune diabetes in NOD mice. **A.** Female NOD mice were injected *i.p.* with 200 µg P40, 100 µg Poly(I∶C), 5 µg LPS from *S. minnesota*, 5 µg Lipid A, 10 µg R848 or PBS once a week, starting at 3–4 weeks of age and for 20 consecutive weeks. Mice were monitored weekly for the advent of glycosuria. Significant prevention from diabetes was observed with most TLR agonists tested (* p<0.05; ** p<0.01) but not with Lipid A. Each panel represents one of 3 independent experiments using 8 mice per group. **B.** A shorter treatment with the TLR2 agonist P40 administered between 4 and 14 weeks also induced significant protection from diabetes (** p<0.01). **C.** Treatment with Poly(I∶C) was highly protective if started up to 10 weeks of age but not later (at 16 weeks of age).

In the spontaneous autoimmune diabetes model, TLR agonists were injected once a week intraperitoneally (*i.p.*) starting at four weeks of age for 20 consecutive weeks. In these conditions, most of the TLR agonists tested were protective (Figure 3A). It is interesting that, for a given TLR, some agonists were not protective although their efficiency at stimulating the TLR pathway *in vitro* has been well demonstrated. Thus, lipid A, which is the lipid portion of LPS, was not protective whereas LPS was (Figure 3A). Most agonists required a long treatment (20 consecutive weeks) for inducing protection, with the exception of P40 which was also protective after a shorter 10-week treatment (Figure 3B). TLR agonists were protective when treatment was started early in disease development, between 4 and 10 weeks of age. A delayed treatment did no longer protect from diabetes (Figure 3C); disease aggravation was never observed.

**Figure 4:**
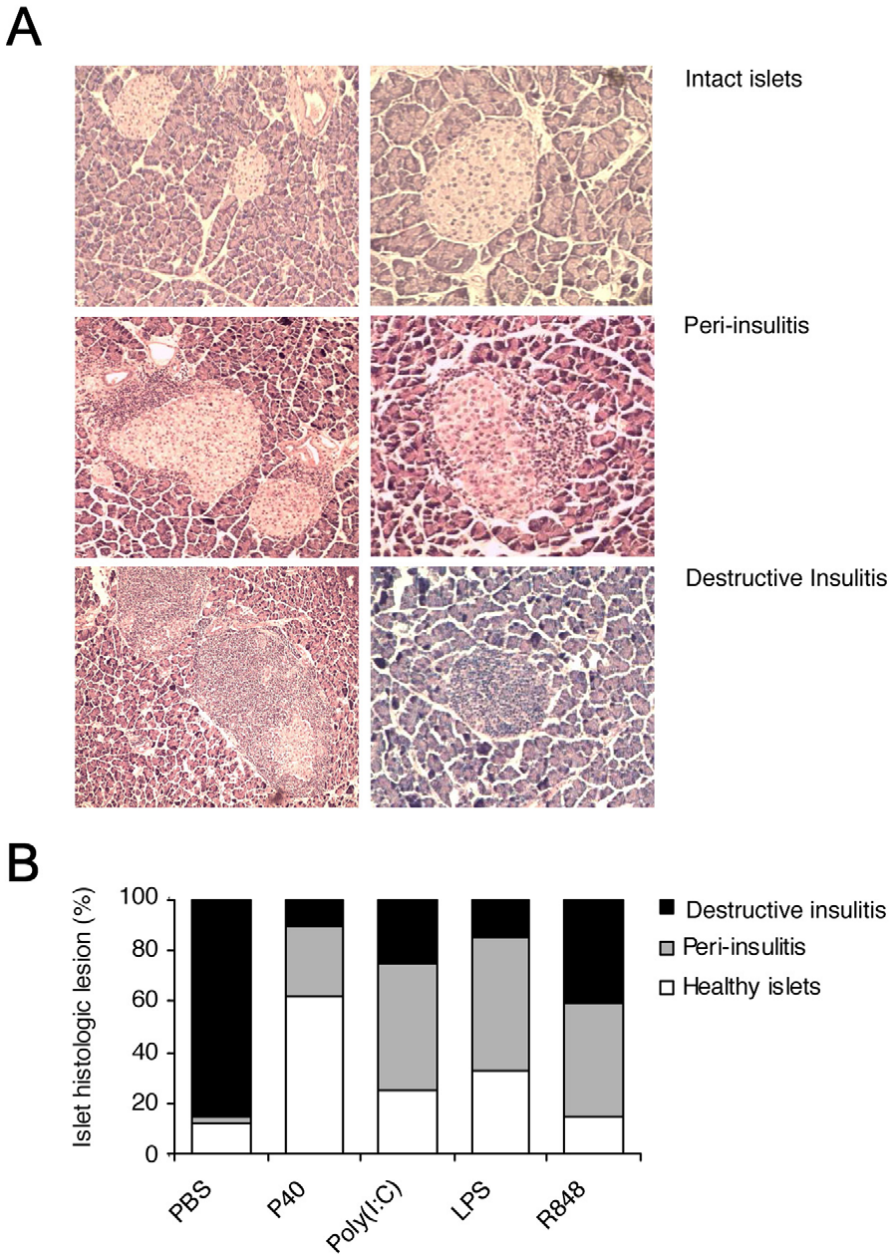
Histological analysis of pancreas. **A.** Representative photomicrographs of distinct patterns observed in pancreas sections. Histological examination of hematoxylin and eosin stained pancreas sections recovered from the various experimental groups was performed (n = 8 per group). Islet infiltration (insulitis) was scored by deducing the proportion of non-infiltrated islets (healthy islets) and of islets showing a non destructive peripheral insulitis (peri-insulitis) or an invasive/destructive insulitis (destructive insulitis). **B.** The relative degree of islet inflammation in mice treated with P40, Poly(I∶C), LPS or R848 is shown in a cumulative histogram as compared to PBS-treated controls.

In protected animals the histological analysis of pancreata showed a reduction in destructive islet infiltration (i.e. invasive insulitis) (Figure 4). It is important to mention here that up to three weeks of age untreated NOD mice do not show any islet infiltration or insulitis. The first infiltrating mononuclear cells appear by 3–4 weeks of age and accumulate up to approximately 12 weeks of age under the form of a non invasive peripheral insulitis (peri-insulitis, see Figure 4A). By 12 weeks of age the topography of the infiltration changes and the insulitis becomes invasive and aggressive (invasive insulitis, see Figure 4A). This form of insulitis is associated to active destruction of insulin-secreting β-cells; this is the point in time where the first mice showing overt hyperglycemia are observed. It appeared that depending on the agonist the effect observed was either a global prevention of the infiltration (in the case of P40) or a control of insulitis progression (in the case of LPS, R848 and Poly(I∶C)) with high proportion of infiltrated islet showing a benign form of non invasive peripheral insulitis (Figure 4). Results on the insulitis patterns in TLR ligand-treated mice versus controls were recovered at the end of the experiments when mice were all aged 25–27 weeks, a quite advanced point in time in disease progression (by 13 weeks after diabetes and invasive insulitis is detected in untreated controls).

### Contrasted effects of MyD88 invalidation on allergic asthma and autoimmune diabetes

**Figure 5:**
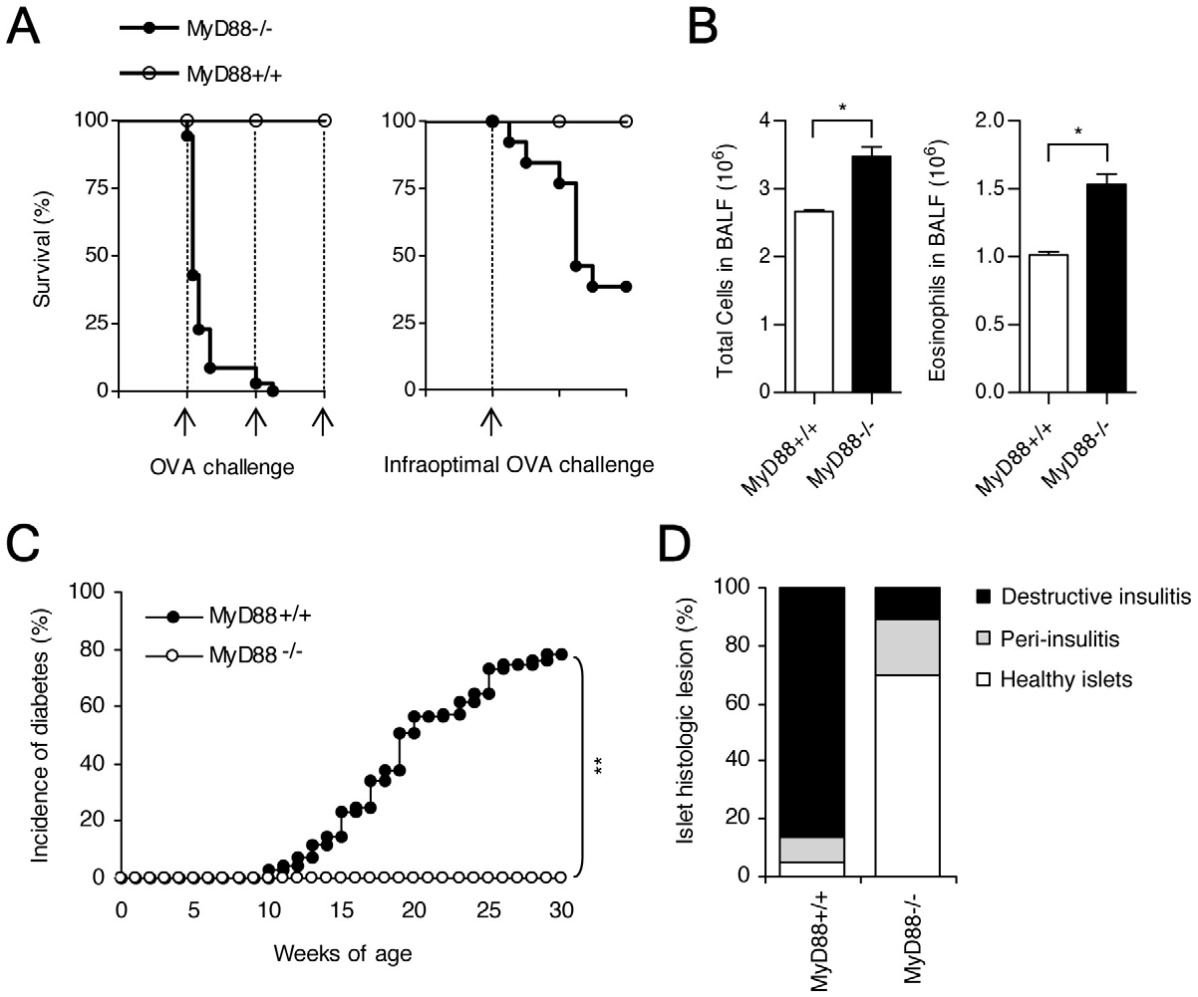
MyD88^**−/−**^ NOD mice are sensitive to airway allergic inflammation but resistant to the development of autoimmune diabetes. As compared to MyD88^+/+^ NOD mice, MyD88^−/−^ NOD mice were hypersensitive to experimental allergic asthma. **A.** The left panel shows the data observed in MyD88^+/+^ (n = 25) and MyD88^−/−^ (n = 35) mice immunized with our conventional protocol, namely immunization with 100 µg of OVA on day 0 and challenge with 50 mg/ml of OVA or NaCl on days 7, 8 and 9. Results are expressed in percentage of survival. The right panel shows the data observed when using an infraoptimal protocol according to which MyD88^+/+^ (n = 10) and MyD88^−/−^ (n = 15) mice were immunized with 50 µg of OVA on day 0 and challenged only once with 20 mg/ml of OVA or NaCl on day 7. Results are also expressed in percentage of survival. **B.** Results show that, using the infraoptimal immunization and challenge protocol, the eosinophil recruitment in BALF was more important in MyD88^−/−^ as compared to MyD88^+/+^ mice (* p<0.05). **C.** Monitoring for the cumulative incidence of spontaneous diabetes showed that MyD88^−/−^ NOD mice (n = 25) were fully protected from disease (littermate female MyD88^+/+^ NOD mice (n = 60) showed a normal disease incidence reaching 80% by 30 weeks of age) (** p<0.01). **D.** Histological examination of hematoxylin and eosin stained pancreas sections recovered from 20-week-old MyD88^+/+^ and MyD88^−/−^ NOD mice (n = 8 per group) showed that a great majority of islets in MyD88^−/−^NOD mice were insulitis free.

In order to study if exogenous or endogenous pathogens (intestinal commensal bacteria) modulate OVA-induced allergic asthma or diabetes, we studied the effect of MyD88 deficiency on these diseases. At variance with results described above which showed a striking parallelism in the pharmacological effects of the TLR agonists in both conditions, impact of MyD88 deficiency on asthma and diabetes was strikingly different. Thus, MyD88^−/−^NOD mice immunized with OVA died (with asphyxia) within few hours following the first or the second aerosol challenge with OVA as compared to wild-type MyD88 sufficient NOD mice which regularly survived the three challenges (Figure 5A). Given these results MyD88^−/−^NOD mice were immunized and challenged with lower doses of OVA (50 µg as compared to 100 µg for immunization and 20 mg/ml as compared to 50 mg/ml for challenge) to allow a significant mouse survival. Results shown on Figure 5B indicate that following this infraoptimal OVA stimulation BALF from MyD88^−/−^ mice contained significantly increased numbers of total leucocytes and eosinophils as compared to controls.

Contrasting with the exacerbated airway allergic response the incidence of autoimmune diabetes was drastically reduced in MyD88^−/−^NOD mice (Figure 5C). None of these mice developed diabetes by 30 weeks of age; a nearly complete prevention of insulitis was also observed (Figure 5D).

### Effect of TLR agonists on immune regulatory pathways

Using both *in vitro* and *in vivo* models we investigated the implication of immune regulatory cytokines and of subsets of regulatory lymphocytes in the protective effect of TLR2, TLR3, TLR4 and TLR7 agonists.

**Figure 6:**
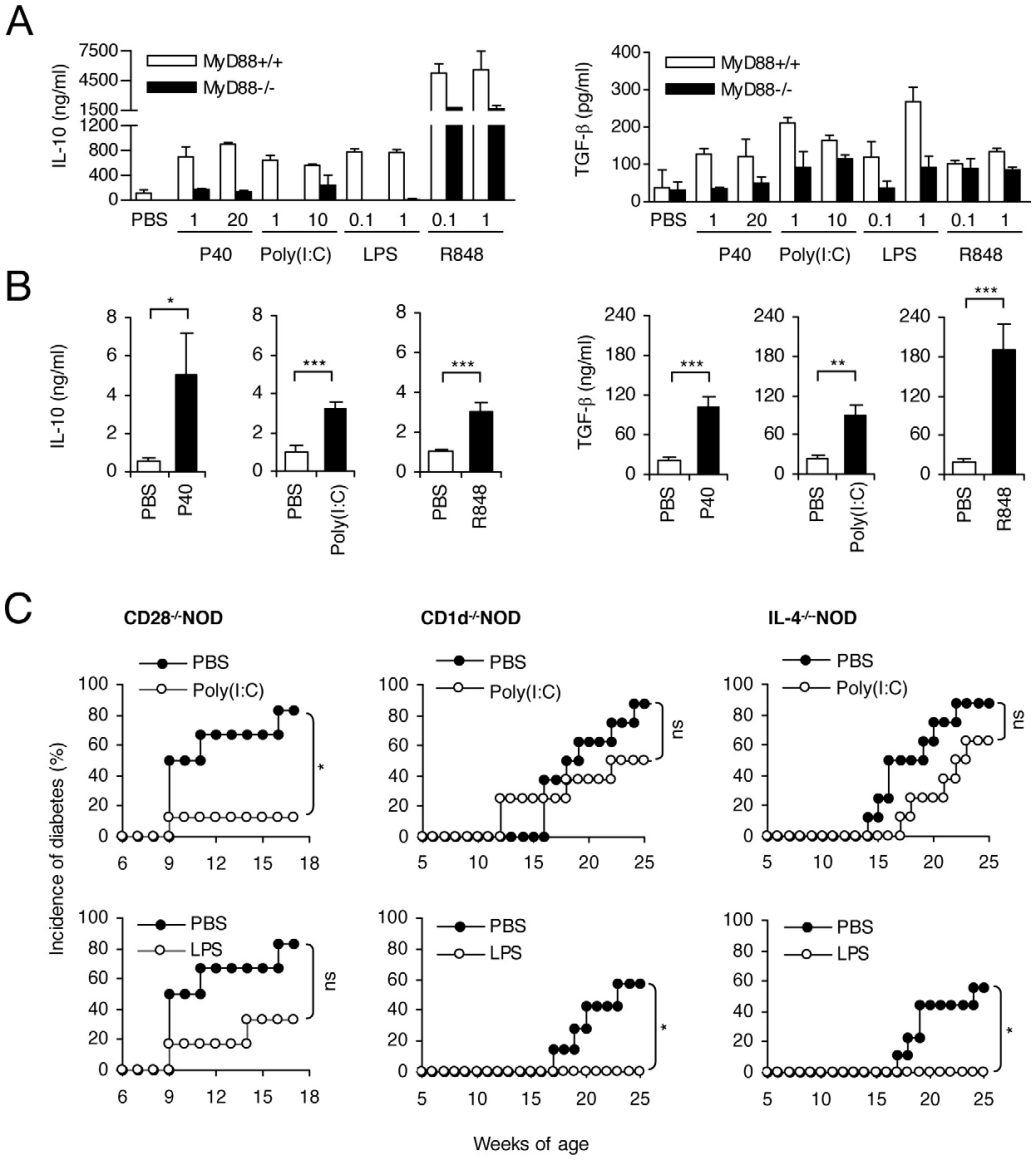
Stimulation of the TLR/MyD88 pathway modulates immune regulatory cytokines and lymphocyte subsets. **A.** *In vitro* stimulation of C57BL/6 mouse spleen cells with varying doses of different TLR agonists (P40, 1 or 20 µg/ml; Poly(I∶C), 1 or 10 µg/ml; LPS, 0.1 or 1 µg/ml; R848 0.1 or 1 µg/ml) induced the production of cytokines such as IL-10 (at 48 hrs) and TGF-β (at 72 hrs). Study of splenocytes from MyD88^+/+^ or MyD88^−/−^ C57BL/6 mice confirmed that the effect is dependent on the MyD88 pathway. Results are expressed as mean cytokine level ± SD. Results are representative of three independent experiments. **B.** Circulating levels of IL-10 and TGF-β were detected following *in vivo* administration of TLR agonists. NOD mice were injected *i.p.* with 20 µg of P40, 100 µg of Poly(I∶C) or 10 µg of R848 (n = 8 per group). Control mice were injected with saline (PBS). Sera were collected 24 hours after the injection and cytokine levels were measured by ELISA (*p<0.05; ** p<0.01; *** p<0.005). **C.** The same conventional treatment protocol (described in Figure 3) with the TLR4 agonist LPS (5 µg/week/mouse) and the TLR3 agonist Poly(I∶C), that protected wild type NOD mice from diabetes, was applied to female NOD mice invalidated for CD28 (CD28^−/−^), CD1d (CD1d^−/−^) and IL-4 (IL-4^−/−^). Results obtained showed that the Poly(I∶C)-induced protective effect was maintained in CD28^−/−^ NOD mice (*p<0.05) but not in CD1d^−/−^ and IL-4^−/−^ NOD mice. As a mirror-like image the LPS-induced protective effect was maintained in CD1d^−/−^ and IL-4^−/−^ NOD mice (* p<0.05) but not in CD28^−/−^ NOD mice. One representative experiment out of 2 performed is shown.

**Figure 7:**
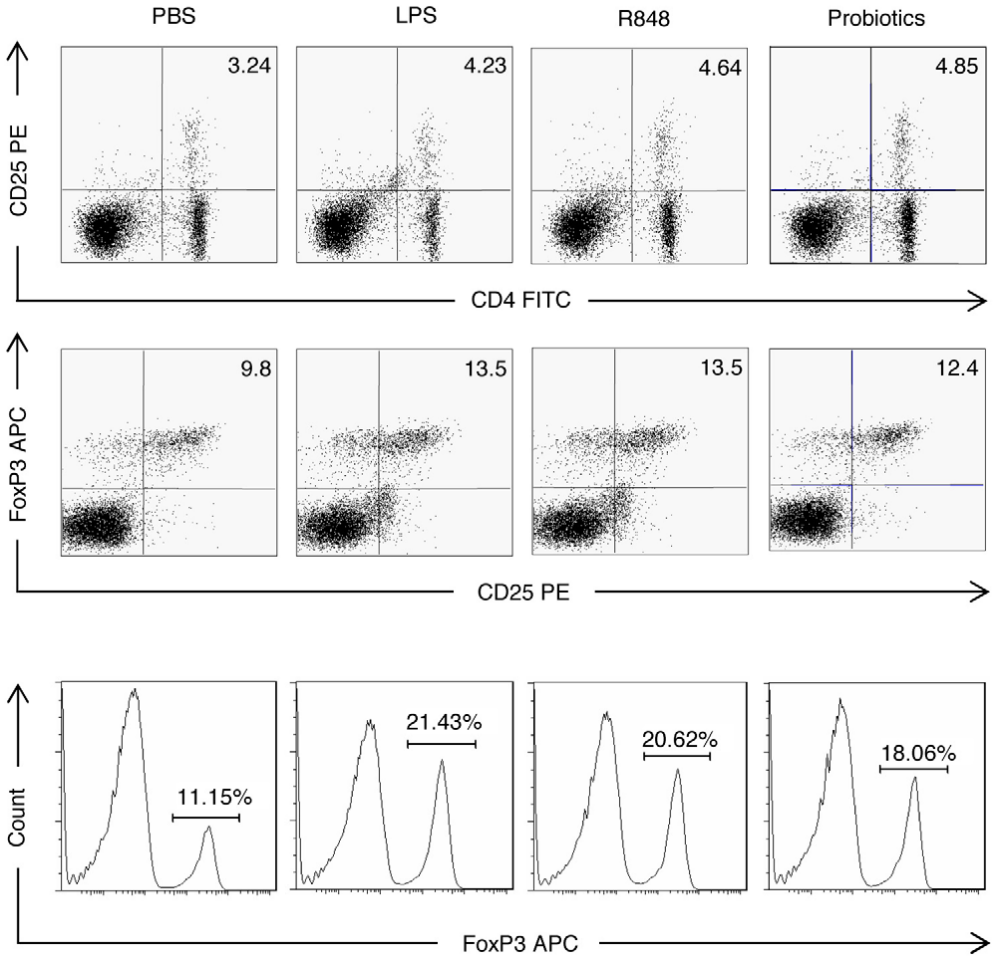
Treatment of NOD mice with TLR4 or TLR7 ligands or probiotics induces CD4^**+**^CD25^**+**^FoxP3^**+**^ Tregs. Mice were injected *i.p.* with 5 µg of LPS, 10 µg of R848 or treated orally with the VSL#3 probiotic preparation 5 days a week for 2 weeks (n = 5 per group). Twenty four hrs after the end of treatment spleen cells were recovered, stained with labeled antibodies specific for CD4, CD25 and FoxP3. Representative flow cytometry plots representing proportions of CD4^+^CD25^+^ and CD25^+^FoxP3^+^ T cells (examined on gated CD4^+^ cells) are shown. In addition, the corresponding histograms showing the total proportions of FoxP3^+^ T cells (within the CD4^+^CD25^+^ and CD4^+^CD25^−^ compartments) are detailed for each experimental group. For the FoxP3 staining, the isotypic controls showed values ranging 0.02–0.09%.

*In vitro*, the 4 agonists induced the production of either TGF-β (for P40, Poly(I∶C) and LPS) or IL-10 (all 4 agonists) by spleen cells (Figure 6A). *In vivo*, serum concentrations of TGF-β andIL-10 were measured 24 hrs after the agonists' injection. As shown in Figure 6B, an increased level of IL-10 and TGF-β was observed after treatment with TLR2, 3 or 7 agonists. We also observed that the administration of a single dose of LPS or R848 increased the number of regulatory CD4^+^CD25^+^Foxp3^+^ T cells in the spleen 24 hrs after injection whereas Poly(I∶C) did not (Figure 7 and data not shown).

To directly evaluate *in vivo* the role of CD4^+^CD25^+^Foxp3^+^ and NKT cells in the protection induced by TLR3 and TLR4 agonists we took advantage of CD28^−/−^ and CD1d^−/−^NOD mice that are deficient in these two subsets respectively. Results shown in Figure 6C demonstrated that the protective effect of LPS was not observed in CD28^−/−^NOD mice but was still present in CD1d^−/−^NOD mice. Conversely, the protective effect of Poly(I∶C) was observed in CD28^−/−^ NOD mice but not in CD1d^−/−^ NOD mice. Interestingly, the TLR3 agonist-mediated protection that was dependent on the presence of NKT cells was abrogated in IL-4^−/−^ NOD mice which was not the case for the protective effect of LPS, whose protective effect did not depend on NKT cells (Figure 6C).

### Effect of probiotic bacteria on allergic asthma and autoimmune diabetes

We studied the capacity of probiotic bacteria to protect from both experimental allergic asthma and autoimmune diabetes.

**Figure 8:**
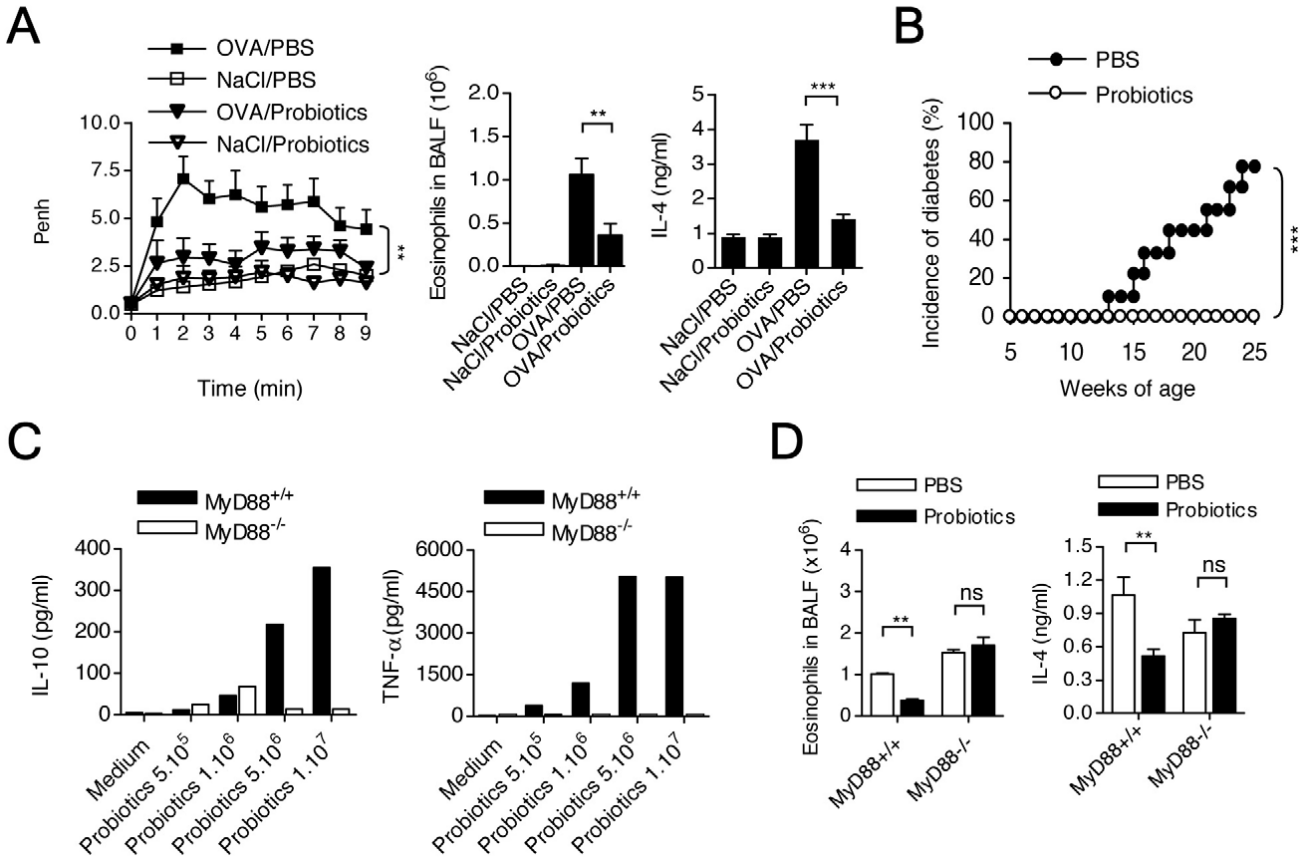
Probiotic administration prevents from both allergic asthma and autoimmune diabetes: a TLR/MyD88 pathway-dependent effect. **A.** NOD mice received 5 days a week for 6 weeks a preparation of probiotics (VSL#3, 5.10^9^ bacteria/mouse) and underwent the conventional OVA immunization/challenge protocol previously described. AHR as well as eosinophil counts in BALF and IL-4 levels in lung homogenates were measured. Results showed that probiotic treatment significantly prevented from experimental allergic asthma (** p<0.01; *** p<0.005). **B.** The same probiotic preparation (VSL#3; 5.10^9^ bacteria/mouse) was administered orally by gavage to female NOD mice three times a week starting at 4 weeks of age (n = 8 per group). Results obtained demonstrated a very significant disease protection (*** p<0.005). **C.** *In vitro* incubation for 24 hrs of peritoneal macrophages from NOD mice with increasing concentrations of the VSL#3 probiotic preparation induced a dose-dependent production of TNF-α and IL-10. The MyD88 dependency of the effect was demonstrated by the lack of effect when macrophages from MyD88^−/−^ mice were analyzed. **D.** The probiotic-induced protection from allergic airway inflammation was MyD88-dependent as illustrated by the comparative results obtained in MyD88^+/+^ mice. The effect was illustrated here by the data on eosinophil counts in BALF showing that MyD88^−/−^ mice (immunized and challenged according to the infraoptimal protocol, see figure 5) were completely refractory to the probiotic-treatment effect as compared to MyD88^+/+^ mice (** p<0.01). Results are representative of one experiment out of two.

In the allergic asthma model we administered VSL#3, a commercial combination of probiotic bacteria (*bifidobacterium, lactobacillium* and *streptococcus*), orally for 6 weeks before the first *i.p.* OVA immunization. In the spontaneous diabetes model, treatment was given once a week for 20 consecutive weeks starting at 4 weeks of age. Results demonstrated significant protection in the two models (Figure 8A,B). The protective effect was dose-dependent (data not shown).

*In vitro*, the same probiotic preparation induced Tumor Necrosis Factor (TNF)- α and IL-10 production by macrophages, an effect that was MyD88-dependent as shown by the absence of production of both cytokines by cells obtained from MyD88^−/−^ mice (Figure 8C).

The TLR dependency of the probiotic bacteria effect was further confirmed *in vivo* in the allergic asthma model since MyD88^−/−^NOD mice were insensitive to treatment, using the low OVA dose described above, compatible with mouse survival (Figure 8D). In MyD88^−/−^NOD mice results showed no effect of probiotic bacteria treatment on either eosinophil recruitment or IL-4 production (Figure 8D).

**Figure 9:**
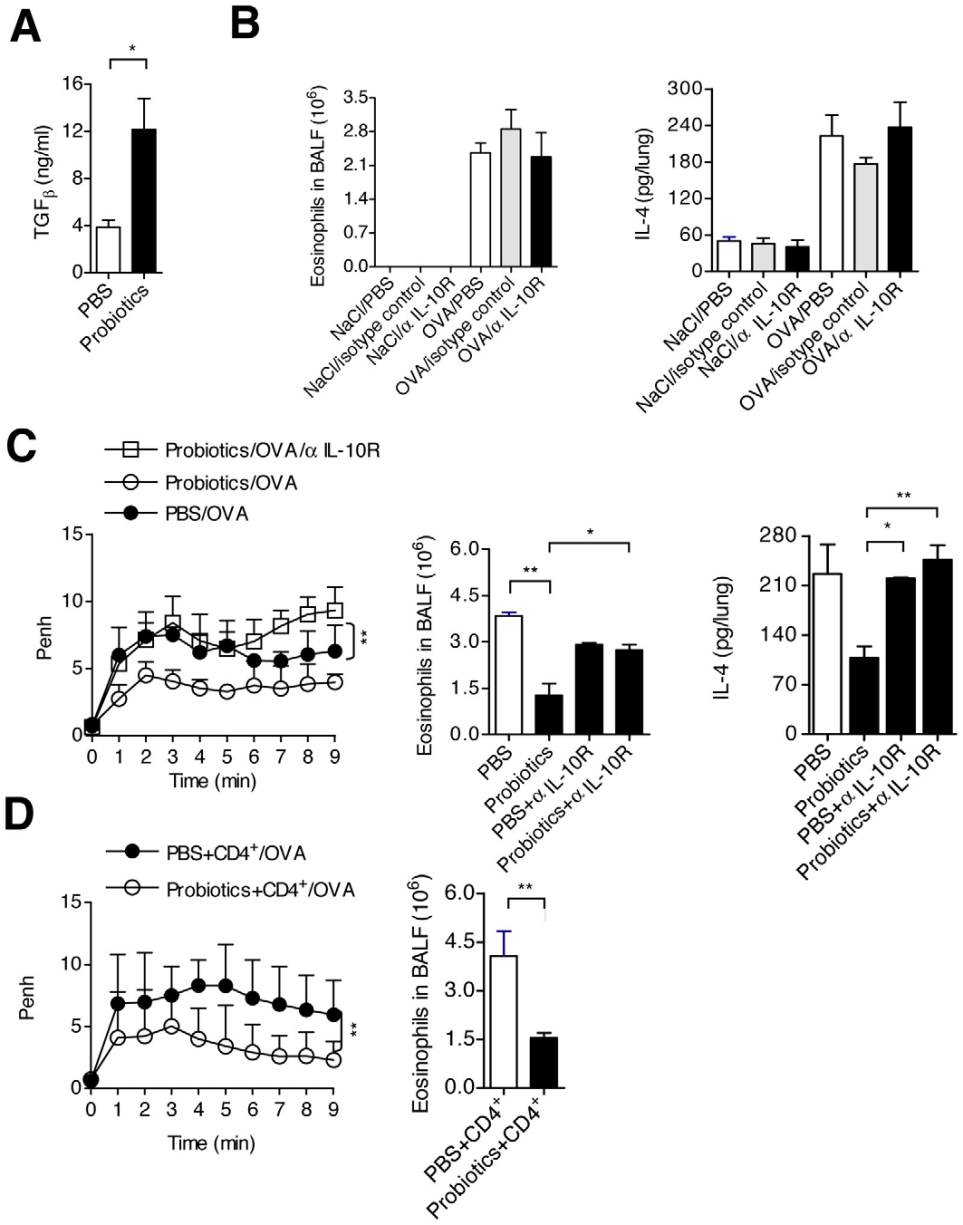
Probiotic treatment and immune regulation. **A.** The VSL#3 probiotic preparation was administered orally to female NOD mice 5 days a week for 2 weeks (n = 5 per group). Twenty four hrs after the last administration sera were collected and circulating TGF-β was measured: increased levels were found in mice treated with the active compound as compared to controls (* p<0.05). **B.** Mice underwent the conventional OVA immunization/challenge protocol previously described and were treated prior to the first OVA challenge with either a neutralizing monoclonal antibody to the IL-10R or an isotype-matched control. Results show that neutralization of IL-10 did not alter allergic inflammation (as assessed by eosinophil recruitment in BALF and IL-4 production). One representative experiment out of two is shown. **C.** NOD mice received 5 days a week for 6 weeks either the VSL#3 probiotic preparation or PBS and then underwent the conventional OVA immunization/challenge protocol previously described. Results obtained showed that the probiotic-protective effect was completely reversed (both in terms of reduction of AHR (** p<0.01) and of eosinophil recruitment in BALF (* p<0.05, ** p<0.01)) following IL-10 neutralization upon administration of an anti-IL-10 receptor monoclonal antibody prior to the first challenge. One representative experiment out of two is shown. **D.** Experimental allergic asthma was induced according to the conventional OVA immunization/challenge protocol already described in normal NOD recipient mice transferred with CD4^+^ cells purified from the spleen of probiotic- or control-treated syngeneic mice. Purified CD4^+^ cells were transferred 24 hrs before the first challenge. Results obtained showed that both AHR and eosinophil recruitment in BALF were significantly decreased (** p<0.01 for both parameters) in recipients of CD4^+^ cells recovered from probiotic-treated donors. One representative experiment out of two is shown.

Interestingly, in the sera of mice protected following probiotic bacteria administration increased levels of TGF-β were detected (Figure 9A). We also detected in the spleen of protected mice an increased frequency of CD4^+^CD25^+^FoxP3^+^ T cells (Figure 7).

In keeping with the *in vitro* data described above the protective effect of probiotic bacteria was IL-10-dependent. In fact, administration of an anti-IL-10 receptor monoclonal antibody before the first aerosol OVA challenge completely abolished the therapeutic effect (Figure 9C).

Finally, the probiotic protective effect was transferable by T cells. Adoptive transfer of CD4^+^ splenocytes from probiotic-treated mice into OVA-challenged wild-type NOD mice significantly inhibited AHR (Figure 9D).

## Discussion

Here we show that a wide spectrum of agonists for various TLRs administered systematically inhibits both allergic and autoimmune responses extending in a comprehensive fashion previous isolated reports. These results are at variance with observations made after using TLR agonists at the site of antigen administration in normal and pathological conditions as discussed in the introduction.

The study of MyD88^−/−^NOD mice provided different results in asthma and diabetes, which initially surprised us in view of the similarity of the effects of all TLR agonists in the two animal models. The acceleration of OVA-induced asthma in MyD88^−/−^NOD mice is in agreement with the pharmacological effects of TLR agonists in this model, corroborating their postulated mode of action and supporting the notion that environmental infectious agents contribute to the modulation of allergic reactions through TLR stimulation. It is important to mention at this point that one should not restrict the discussion to the MyD88 adaptor. Other important adaptor molecules have been identified such as TRIF that is involved in TLR4 and TLR3 signaling [39] and which may impact in the effect we observed following LPS and/or Poly (I∶C) treatment remains to be determined.

These data are in keeping with the previous report showing that MyD88^−/−^C57BL/6 mice present a Th2 skewed balance with increased IgE levels [40]. It was more difficult to explain the complete prevention of diabetes observed in MyD88^−/−^NOD mice reported by Chervonsky and observed in our study [36]. The first interpretation is that MyD88 is mandatory for diabetes development. Another possibility is that MyD88 deficiency favors a significant change in the composition of the commensal flora or the development of infections that both inhibit diabetes onset [36], [41]. The second interpretation is supported by Chervonsky's data showing that specific pathogen-free (SPF) MyD88^−/−^NOD mice are protected from diabetes (as in our own study) but germ-free MyD88^−/−^NOD mice show normal diabetes incidence [36]. It may be interesting to mention here that invalidation of the CD28 gene also shows contrasting effects on diabetes and asthma with acceleration of diabetes and reduction of asthma [42], [43]. Finally, it appears that our results showing diabetes prevention with TLR agonists provide further support for TLR involvement in the control of the diabetogenic response in NOD mice.

Our results point to several non mutually exclusive mechanisms underlying asthma and diabetes prevention by TLR agonists. These include: 1) the production of immune regulatory cytokines that was observed both *in vitro* and *in vivo*, 2) the involvement of CD4^+^CD25^+^FoxP3^+^ regulatory T cells indicated by the loss of protection observed in CD28^−/−^ NOD mice that are devoid of CD4^+^CD25^+^T cells [44]. A word of caution is however needed concerning CD28 invalidation as it may alter a number of other immune parameters than the production/differentiation of CD4^+^CD25^+^FoxP3^+^ regulatory T cells and 3) the involvement of NKT cells demonstrated by the absence of diabetes protection in CD1d^−/−^NOD mice that are deprived of NKT cells [45]. It is interesting to speculate at this point that, independently form the precise mechanism(s) mentioned above involved in the ‘induction’ phase of the TLR ligand therapeutic effect and which *per se* are not antigen-specific, over long-term ‘maintenance’ of protection from allergy and autoimmunity may involve *bona fide* immune tolerance i.e an antigen-specific effect. Among the TLR agonists protective for diabetes, it is interesting to highlight that the regulatory mechanisms involved differed. Thus, TLR3 stimulation by Poly(I∶C) required the presence of NKT cells but not that of CD4^+^CD25^+^FoxP3^+^ T cells. Conversely, TLR4 stimulation by LPS required the presence of CD4^+^CD25^+^FoxP3^+^ T cells but not that of NKT cells. It is also striking that diabetes protection by TLR3 stimulation required the presence of IL-4 which was not the case for TLR4-induced protection. It is to note that the role of CD4^+^CD25^+^ T cells could not be tested in the allergic asthma model since CD28^−/−^ mice do not develop OVA-induced asthma [43].

Finally, the role of cytokines is difficult to associate with a given TLR since TLR-induced cytokine production, which was consistently observed, varied with each agonist: one should note however that TLR2, TLR3 and TLR7 agonists preferentially stimulated IL-10 and TGF-β production which was not the case for the TLR4 agonist LPS. More generally these data emphasize the complexity and the importance of TLR-mediated stimulation of immunoregulatory cytokines as also recently shown by S. Cohen et al. in experimental allergic encephalomyelitis [46].

In the context of this study we were faced with a dilemma between attempting to reproduce ‘real life’, which means using intact microorganisms or their extracts, or using a more reductionist and contrived approach taking advantage of well defined components of the microorganisms, such as the TLR ligands we discussed, to get further insights into the fine cellular and molecular mechanisms underlying the protective effects. Therefore, we conducted the studies discussed above using well defined TLR ligands in parallel to probiotics which we selected among other microorganisms inasmuch as they have been shown to have a significant therapeutic effect in clinical atopic dermatitis [47]–[49].We provide evidence showing that probiotics function through TLR stimulation as first, *in vitro* probiotics failed to induce cytokine production by spleen cells from MyD88^−/−^ NOD but not from wild-type NOD mice and second, they did protect MyD88^−/−^ NOD from allergic asthma in clear contrast with the significant therapeutic effect observed in wild-type NOD mice. This model allowed us to perform complementary mechanistic studies that showed tranfer of protection by CD4^+^ T cells, stimulation of IL-10 production and loss of the protective effect following administration of a neutralizing anti-IL-10 receptor antibody.

The association of a defined mechanism with a specific TLR is further complicated by the fact that for a given TLR distinct agonists do not show the same pharmacological profile: for instance in the case of TLR4, LPS is protective whereas lipid A is not. In our initial screening of various TLR ligands it appeared that lipid A, as compared to LPS, had a lower capacity to induce cytokine production. This ‘lower’ stimulating capacity might be the explanation for the lack of ‘therapeutic’ activity of this TLR4 ligand. In any event these data indicates that all ligands of a given TLR do not have the same protective effect paving the way for an immunopharmacology of each individual TLR ligand. Concerning the molecular basis for these differential behavior, as mentioned above, it has been well established that different adaptor proteins i.e. MyD88 and TRIF mediate TLR4 signaling [39]. One may speculate that signaling by given TLR4 ligands may differentially involve these adaptors.

The whole of these data pave the way for a new pharmacology of TLR stimulation in allergy and autoimmunity with contrasting effects depending not only on the nature of the TLR receptor but also on that of the specific ligand.

If it were confirmed that TLR stimulation modulates the function of regulatory CD4^+^CD25^+^FoxP3^+^ T cells or NKT cells, it would be of central importance to determine whether this effect is direct or indirect (e.g. through dendritic cells) [50], [51]. Studies from different laboratories indicate the presence of TLRs on various subsets of regulatory T cells with however some contradictory data [52]–[54]. One should also note that mechanisms other that those discussed above could operate notably IL-10 production by B cells [55]–[58]

The relevance of these results for the evolving epidemiology of asthma and autoimmune type 1 diabetes is intriguing for the search of new preventive treatments of these diseases. Results presented in this manuscript indicate that TLR ligands present in infectious agents could contribute to the protection afforded by these agents against these diseases according to the hygiene hypothesis. At the therapeutic level, our results suggest the possibility of using TLR ligands or probiotics in the prevention of allergic and autoimmune diseases inasmuch safety is fully documented. The clinical relevance of the approach described is based on the widely accepted concept that in the future major breakthrough in the management of these diseases will come from prevention rather than treatment of established disease. This approach has already been implemented in atopic dermatitis, using probiotics, and insulin-dependent type 1 diabetes in subjects at risk of developing the disease (children with family history of disease and, when available as in insulin-dependent diabetes expressing at risk genetic alleles and/or autoantibody markers) using candidate autoantigens in particular insulin delivered orally, parenterally or intranasally [59], [60]. In both situations these studies involved long-term daily treatments (several months) as in our present studies. It is important to mention with regard to our own study that the promising but still to be confirmed encouraging results obtained with probiotics in atopic dermatitis [47]–[49]. One would certainly prefer, in the future, to use well-defined chemical synthetic TLR agonists as those used in our work. Probiotics present the interest of direct accessibility and low toxicity but suffer from poor standardization. TLR agonists are probably more potent but are confronted with the potential risk of stimulating undesirable immune responses which would necessitate further safety studies.
